# Influence of yoga on mood states, distress, quality of life and immune outcomes in early stage breast cancer patients undergoing surgery

**DOI:** 10.4103/0973-6131.36789

**Published:** 2008

**Authors:** Raghavendra M Rao, H R Nagendra, Nagarathna Raghuram, C Vinay, S Chandrashekara, K S Gopinath, B S Srinath

**Affiliations:** Department of Yoga Research, Swami Vivekananda Yoga Anusandhana Samsthana, Bangalore, India; 1 Department of Clinical Immunology, M.S Ramiah Medical Teaching Hospital, Bangalore, India; 2 Department of Surgical Oncology, Bangalore Institute of Oncology, Bangalore, India

**Keywords:** Cancer, immunity, mood, surgery, yoga

## Abstract

**Context:**

Breast cancer patients awaiting surgery experience heightened distress that could affect postoperative outcomes.

**Aims:**

The aim of our study was to evaluate the effects of yoga intervention on mood states, treatment-related symptoms, quality of life and immune outcomes in breast cancer patients undergoing surgery.

**Settings and Design:**

Ninety-eight recently diagnosed stage II and III breast cancer patients were recruited for a randomized controlled trial comparing the effects of a yoga program with supportive therapy plus exercise rehabilitation on postoperative outcomes following surgery.

**Materials and Methods:**

Subjects were assessed prior to surgery and four weeks thereafter. Psychometric instruments were used to assess self-reported anxiety, depression, treatment-related distress and quality of life. Blood samples were collected for enumeration of T lymphocyte subsets (CD4 %, CD8 % and natural killer (NK) cell % counts) and serum immunoglobulins (IgG, IgA and IgM).

**Statistical Analysis Used:**

We used analysis of covariance to compare interventions postoperatively.

**Results:**

Sixty-nine patients contributed data to the current analysis (yoga *n* = 33, control *n* = 36). The results suggest a significant decrease in the state (*P* = 0.04) and trait (*P* = 0.004) of anxiety, depression (*P* = 0.01), symptom severity (*P* = 0.01), distress (*P* < 0.01) and improvement in quality of life (*P* = 0.01) in the yoga group as compared to the controls. There was also a significantly lesser decrease in CD 56% (*P* = 0.02) and lower levels of serum IgA (*P* = 0.001) in the yoga group as compared to controls following surgery.

**Conclusions:**

The results suggest possible benefits for yoga in reducing postoperative distress and preventing immune suppression following surgery.

Awaiting surgery is a distressing experience for most breast cancer patients.[1–3] It has been described as a acute, short-term stressor with multiple stressful components such as concerns regarding one's physical condition, postoperative recovery, hospital admissions, anticipating painful procedures, image problems, confronting cancer diagnosis and worries about survival and recovery.[4] It is known that such concerns evoke strong emotional and psychological reactions in the subjects further heightening preoperative distress. Such heightened preoperative distress has been found to be related to longer hospital stays, delayed recovery, more postoperative complications, pain and increased need for medications.[4,5] These distressing symptoms are also known to impair local and systemic immune responses leading to delayed wound repair[6,7] and postoperative infections.[8,9] Apart from direct effects on endocrine and immune function, greater pain sensitivity and distress of more anxious patients may affect recovery because of reduced compliance; for example, breathing exercises reduce the risk of pneumonia following surgery.[10] Patients may become more cautious about following recommendations for walking, breathing or coughing because of pain and distress further affecting the process of recovery.[5]

A multitude of factors seem to affect antitumor and innate immune responses collectively during surgical recovery such as mood, perceived stress, anti-inflammatory analgesics and anesthesia. Medications that reduce pain and distress such as analgesics,[11] corticosteroids, morphine[12] and anesthetics[13,14] following surgery can directly help reduce treatment-related distress, improve functional well being and reduce consequent immune suppression. However, such medications are not always cost-effective and have side effects ranging from gastrointestinal distress, nausea and, sleep disturbances[10] and also excessive use can dampen both innate and anti-tumor immune responses.[4] For example, marked suppression of NK cell activity and count is observed following surgical procedures that often last for an extended period inspite of the use of anti-inflammatory and anesthetic medications.[15] This transitory dysfunction of NK cells may create a favourable milieu for metastases. This has been attributed to loss of control over dormant micrometastases or from an inability to destroy tumor cells released perioperatively.[16] Moreover, changes in NK cell activity and number seem to be affected by mood states with decreased NK cell numbers seen with depressed mood[17,18] and decreased NK cell activity is related to anxiety states.[19]

There is evidence to show that interventions that alter appraisal, coping and/or mood may also modulate immune and endocrine function, thereby enhancing surgical recovery.[20,21] Even modest interventions that have relatively small consequences for psychological distress such as educating the patient about surgery[22] and improving the ambience in the wards[23] are known to influence the recovery process. Several meta-analyses of presurgical intervention studies have argued that association between presurgical intervention and clinical outcome is clinically meaningful.[24] Depending on the meta-analysis, two thirds to three quarters of intervention patients had better outcomes than control subjects with the size of improvement ranging from 20–28%.[24] These stress reduction and behavioral interventions apart from reducing distress, are also known to affect immune responses in general.[25,26]

Yoga is one such psychotherapeutic intervention, which, has been used effectively in numerous health care conditions where stress was believed to play a role. General effects of yoga in promoting health are due to its ability to establish stable autonomic balance,[27] development of hypometabolic states,[28] improvement of physical efficiency,[29] improvement of thermoregulatory efficiency,[30] increase in cardiopulmory functions,[31] improved immunological tolerance,[32] neuro-endocronine functions,[33–35] improved mood states[36–38] and a tranquil state of mind to combat stress.[39] These physiological benefits possibly explain the rationale for using yoga intervention to reduce distress and improve immune outcomes in cancer subjects undergoing surgery. Moreover, various techniques of yoga have shown to improve mood states, reduce stress, improve quality of life and, adjustment in cancer patients.[40] In an earlier study, we have shown yoga to reduce treatment-related distress and symptoms in breast cancer patients undergoing chemotherapy.[41] This may have implications in the current context wherein high psychological distress is seen preoperatively. We hypothesize that yoga interventions would help reduce psychological distress and improve anti-tumor immune responses in breast cancer patients following surgery.

## SUBJECTS AND METHODS

This is a single center, randomized controlled trial which recruited ninety-eight recently diagnosed stage II and III operable female breast cancer patients to evaluate the effects of yoga intervention versus a supportive therapy and exercise rehabilitation. Time following diagnosis ranged from 1–4 weeks. Cancer staging was done using the international union against cancer staging system. Participants were recruited between January 2000 to June 2004 in a comprehensive cancer care center in Bangalore. The study was approved by the ethical committee of the recruiting cancer center. Patients were included if they met the following criteria: i) women recently diagnosed with operable breast cancer, ii) age between 30 to 70 years, iii) Zubrod's, performance status 0–2 (ambulatory > 50% of time), iv) high school education iv) willingness to participate v) primary treatment as surgery. Patients were excluded if they had i) a concurrent medical condition likely to interfere with treatment, ii) any major psychiatric, neurological illness or autoimmune disorders, iii) secondary malignancy iv) presenting with infections or history of recent infections in the past month. The details of the study were explained to the participants and their informed consent was obtained.

**Figure 1:**
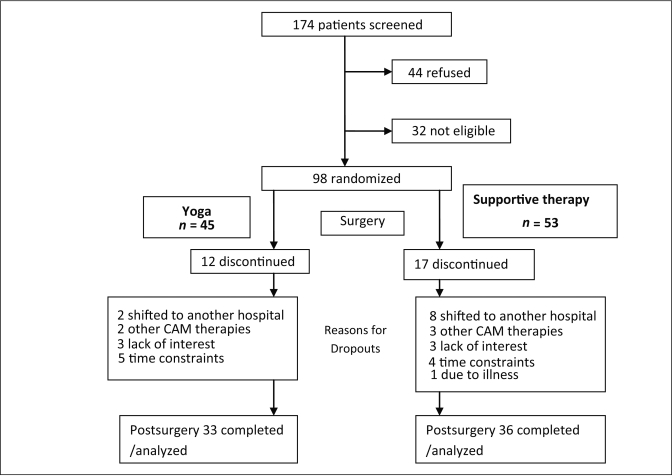
Trial profile

Baseline assessments were done on 98 patients prior to their surgery. Sixty-nine patients contributed data to the current analyses at the second assessment (postsurgery four weeks after surgery). The reasons for dropout were attributed to migration to other hospitals, use of other complementary therapies (*e.g.* Homeopathy or Ayurveda), lack of interest, time constraints and other concurrent illness [Figure 1].

At the initial visit before randomization, investigative notes and standard self-report questionnaires assessing anxiety, depression and quality of life were used to get demographic information, medical history, clinical data, intake of medications during their hospital visit. About 12 ml of blood samples were collected in vacuettes under sterile conditions on the day of their surgery. Blood samples were collected between 8 a.m. to 12 p.m. for all participants to reduce diurnal variability. Follow-up assessments were done at four weeks following surgery before the commencement of any adjuvant treatment.

### Randomization

Subjects consenting to participate in this study were randomly allocated to receive either yoga (intervention) or supportive therapy plus exercise therapy prior to their surgery using random numbers generated by a random number table. Randomization was performed using opaque envelopes with group assignments, which were opened sequentially in the order of assignment during recruitment with names and registration numbers written on their covers. Yoga being a popular intervention, it was not possible to mask the yoga intervention from the subjects although they were initially told that they would be participating in a postoperative rehabilitation program. However, the investigators (treating surgical oncologists) were blind to the intervention.

### Measures of stress

Stress was assessed using standard self-report questionnaires such as the State Trait Anxiety Inventory (STAI)[42] for anxiety and Beck's Depression Inventory (BDI)[43] for depression.

STAI consists of separate self-report scales for measuring two distinct anxiety concepts: state anxiety and trait anxiety, each having twenty statements. The respondents are required to rate themselves on a four point scale: ‘not at all to very much so’ on various anxiety-related symptoms which they experience. This has been used widely in earlier studies on cancer populations and with a concurrent validity ranging from 0.75 to 0.80 with other tests.

Beck's Depression Inventory is a self-report measure used to assess behavioral manifestations of depression. The inventory is composed of 21 categories of symptoms and attitudes, each with a graded series of 4–5 evaluation statements ranked to indicate the range of severity of symptoms from neutral to maximal severity. This instrument has a reliability of 0.48–0.86 and validity of 0.67 with the Diagnostic and Statistical Manual of Mental Disorders (DSM) diagnostic criteria for depression.

### Measures of quality of life and stress symptoms

Quality of life of study participants was ascertained using the Functional Living Index of Cancer (FLIC).[44] This scale is a self-administered measure of the global quality of life for cancer patients having a high correlation (0.44–0.75) with other scales.

A subjective symptom checklist was developed during the pilot phase to assess stress, treatment-related side effects, problems with sexuality and image and relevant psychological and somatic symptoms related to breast cancer. The checklist consisted of 31 such items, each evaluated on two dimensions, the severity graded from “none to very severe (0–4)” and distress from “not at all to very much (0–4)”. This scale measured the total number of symptoms experienced, total and mean severity and distress score and was evaluated previously in a similar breast cancer population.[45]

### Immune assays

Blood samples were collected in three separate vaccutainers: 7 ml of each blood sample was collected in a heparinized vacuette for the separation of peripheral blood lymphocytes, 2 ml in a sodium citrate vacuette for plasma and 2 ml in a plain vacuette for serum.

Blood cell separation: Peripheral Blood Lymphocytes were isolated from 7 ml of heparinized blood using ficoll gradients (Histopaque 1077 R, Sigma Inc.). The isolated lymphocytes were then washed in phosphate-buffered saline (PBS), treated with 4% glacial acetic acid to lyse the red blood cells (RBCs) and again washed with PBS. The lymphocytes were then counted on a hemocytometer/ coulter counter and diluted at concentration of 50,000 cells/ml in Tris-buffered saline (TBS). Thereafter, the cell suspension was centrifuged and the cells fixed on the slides using an acetone-methanol medium. The NK Cell count was determined by immunohistochemistry using the standard Alkaline Phosphatase Anti-Alkaline Phosphatase technique (APAAP). Briefly, the cells fixed on the slides were treated with an anti-CD56 antibody (DAKO Cytomation) and then with a secondary antibody conjugated with APAAP. Cells with the CD56 surface antigen bound to these antibodies and took up the fast red stain giving a red glow over the periphery. The cells were counterstained with hematoxylin. Only those cells which took up the fast red stain and had pink to red stained edges were counted as CD56-positive cells as against others, which took up only hematoxylin and appeared blue. The cells were counted in two hundred fields and the mean percentage of CD56-positive cells per hundred fields extrapolated. Serum Immunoglobulins (IgG, IgM and IgA) were assessed using an Immunoturbidometry assay with an autoanalyzer.

### Interventions

The intervention group received an “integrated yoga program” and the control group received “supportive counseling and exercise rehabilitation.” While the goals of the yoga intervention were stress reduction and improvement in shoulder mobility, the goals of the control intervention were to reinforce social support and prevent shoulder restriction. The yoga intervention consisted of a set of breathing exercises, *pranayama* (voluntarily regulated nostril breathing) and yogic relaxation techniques. These practices were based on the principles of attention diversion and relaxation to cope with day-to-day stressful experiences. These sessions were administered by an instructor at the subjects' bedside prior to surgery and during their postoperative recuperation in the hospital. Following their discharge, subjects were asked to practise at home for the next four weeks. Subjects were also provided audiotapes of an instructor's voice to help them practise at home so that a familiar voice could be heard on the cassette. Their practice was monitored on a day-to-day basis by their instructor through telephone calls once a week. Subjects were encouraged to maintain a daily log listing the yoga practices done, use of audio-visual aids for practice, duration of practice, experience of distressing symptoms, intake of medication and diet history.

Supportive counseling sessions as control intervention included two important components: “i) education and reinforcing social support and ii) shoulder exercise for postoperative rehabilitation.” We chose to have this as a control intervention to prevent shoulder restriction and to control for any nonspecific effects of the yoga program that may be associated with adjustment such as attention, support and a sense of control. Moreover, these interventions have been shown to hasten recovery from surgery in earlier studies.[22,24]

Subjects and their caretakers were invited to participate in an introductory session lasting 60 minutes before surgery where they were given information about surgery and management of its related side effects, taught shoulder exercises and mobilization by the physiotherapist and provided information about a variety of common questions. The interventions were imparted at the patient's bedside and subjects were asked to perform the shoulder exercises at their home following their discharge until they receive adjuvant therapy. Both groups received four such in-person sessions of intervention during their hospital stay.

### Data analysis

Data was analyzed using SPSS 10.0 for windows. Data was tested for normality and homogeneity. An analysis of covariance was done on all assessments 3–4 weeks postsurgery using their respective baseline (presurgery) measures as covariates. The within-groups effects was analyzed using a paired t test.

## RESULTS

The age, stages of disease, grade and node status were similar in the yoga and supportive therapy (control) groups [Table 1]. The mean years of education of the study sample was 12.49 ± 2.67 years, with a minimum of seven years and a maximum of 17 years of education. The mean overall age of the subjects was 49.2 ± 9.6 years in both groups. All subjects had adequate nutritional status with the majority of them having a body mass index (BMI) between 19–25 (57%) and the rest above 25 (43%).

**Table 1:** Demographic characteristics

	All subjects		Yoga group		Control group	
			
	*n*	(%)	*n*	(%)	*n*	(%)
Stage of breast cancer						
II	31	45	17	54.83	14	45.16
III	38	55	16	42.1	22	57.89
Grade of breast cancer						
I	1	1	1	100	0	0
II	8	12	6	75	2	25
III	60	87	26	43	34	57
Menopausal status						
Pre	33	48	20	61	13	39
Post	33	48	11	33	22	67
Peri	1	1	1	100	0	0
Posthysterectomy	2	3	1	50	1	50
Type of surgery						
Mastectomy	52	75.4	24	53.8	28	46.2
Breast conservation	17	24.6	9	47.1	8	52.9
Stressful life events in the past two years						
Yes	19	28	8	42	11	58
No	50	72	25	50	25	50

Control group: supportive therapy group

### Psychological outcomes

#### Measures of mood [Table 2]

**Table 2:** Comparison of anxiety state and trait, depression and quality of life scores in yoga and control groups following surgery using paired t test and ANCOVA

Outcome measures	Presurgery Y (*n* = 33), C (*n* = 36)	Postsurgery
STAI-Anxiety state Scores		
Yoga		
Mean ± SD (standard deviation)	44.2 ± 10.9	34.0 ± 3.9**
Control		
Mean ± SD	49.6 ± 12.0	38.4 ± 8.1
⇕Adjusted mean (y-c)		-2.79
(95% CI)		-5.5 to 0.079
STAI-Anxiety Trait Scores		
Yoga		
Mean ± SD	42.8 ± 9.8	33.4 ± 4.4**
Control		
Mean ± SD	47.4 ± 11.6	40.3 ± 8.7
⇕Adjusted mean (y-c)		-4.24**
(95% CI)		-7.0 to -1.4
Beck's Depression Scores		
Yoga		
Mean ± S.D	12.1 ± 6.4	11.6 ± 4.5
Control		
Mean ± S.D	15.1 ± 7.3	15.1 ± 5.3
⇕Adjusted mean (y-c)		-2.63**
(95% CI)		-4.6 to -0.65
FLIC Quality of Life Scores		
Yoga		
Mean ± S.D	109.8 ± 21.5	107.6 ± 13.1**
Control		
Mean ± S.D	100.7 ± 17.4	92.7 ± 17
⇕Adjusted mean (y-c)		
(95% CI)		11.82***
		5.1 to 18.5

* *P* < 0.05

** *P* < 0.01

*** *P* < 0.001, y = Yoga, c = Control/ Supportive therapy group

*P* values for paired † test in yoga and control groups

⇕ Posttest scores (y-c) adjusted for their baseline scores between yoga and control groups with 95% CI and using ANCOVA for *P* values

Participants reported high levels of anxiety at baseline (before surgery). A paired samples t test done to assess the changes in anxiety state following surgery within groups showed a significant decrease in anxiety state following surgery in both control [t (35) = 6.69, *P* < 0.001, 95% confidence interval, CI (4.9 to 10.6)] and yoga groups [t (32) = 6.41, *P* < 0.001, 95% CI (7.6 to 14.3]. Analysis of covariance using baseline anxiety states as a covariate showed a significant decrease in anxiety states following surgery [F (66) = 4.22, *P* = 0.04, 95%CI (-5.6 to -0.3)], in the yoga group as compared to controls. STAI trait scores were initially high in the period between diagnosis and surgery. A paired samples t test done to assess the changes in the anxiety trait following surgery within groups showed a significant decrease in the anxiety trait following surgery in both control [t (35) = 5.50, *P* < 0.001, 95% CI (4.9 to 10.6)K ] and yoga groups [t (32) = 6.1, *P* < 0.001, 95% CI (5.8 to 11.7) ]. ANCOVA using baseline anxiety trait score showed significant decrease in the anxiety trait scores following surgery, [F (66) = 9.8, *P* = 0.002, 95% CI (-7.2 to -1.7)]. There was no significant within-group differences following surgery in the depression scores. Analysis of covariance using baseline depression scores as a covariate showed a significant decrease in depression following surgery [F (66) = 7.6, *P* = 0.008, 95% CI (-4.6 to -0.73)].

#### Measures of quality of life and stress symptoms [Table 3]

**Table 3:** Comparison of number, severity and distress of symptoms on symptom checklist in yoga and control groups following surgery using paired t test and ANCOVA

Outcome measures	Presurgery Y (*n*= 33), C (*n*= 36)	Postsurgery
Number of Symptoms		
Yoga		
Mean ± (S.D)	8.2 ± 3.5	7.5 ± 2
Control		
Mean ± (S.D)	9.1 ± 4.2	8.8 ± 2.5
⇕Adjusted mean (y-c)		
(95% CI)	-2.79	-5.5 to 0.079
Severity of symptoms		
Yoga		
Mean± (S.D)	11.7 ± 6.7	10.8 ± 3.6
Control		
Mean± (S.D)	15.6 ± 8.6	15.3 ± 5.1
⇕Adjusted mean (y-c)		
(95% CI)		-4.24**
		-7.0 to -1.4
Symptom distress		
Yoga		
Mean (S.D)	14.4 ± 8.7	11.5 ± 4.2*
Control		
Mean (S.D)	16.9 ± 9.8	16.3 ± 6.0
⇕ Adjusted mean (y-c)		-2.63**
(95% CI)		-4.6 to -0.65

* *P* < 0.05

** *P* < 0.01

***P < 0.001, y = Yoga, c = Control/ Supportive therapy group

*P* values for paired t test in yoga and control groups

⇕ Posttest scores (y-c) adjusted for their baseline scores between yoga and control groups with 95% CI and using ANCOVA for *P* values

A paired samples t test showed a significant decrease in distress in the yoga group alone following surgery [t (32) = 2.1, *P* = 0.05, 95% CI (0.006 to 5.7)]. There was a significant decrease in symptom severity [F (66) = 12.8, *P* = 0.001, 95% CI (-5.1 to 1.4)] and distress [F (66) = 13.6, *P* <; 0.01, 95% CI (-6.3 to -1.8)] in the yoga group as compared to the controls following surgery. There was no significant within-group differences following surgery in the quality of life scores. Analysis of covariance using baseline quality of life scores as a covariate showed significant improvements in quality of life following surgery [F(66) = 12.34, *P* = 0.01, 95% CI (4.7 to 19.8)] in the yoga group as compared to the controls.

### Immune measures

#### Serum immunoglobulins [Table 4]

**Table 4:** Comparison of serum immunoglobulins IgG, IgA and IgM levels (g/L) in yoga and control groups following surgery using paired t test and ANCOVA

Outcome measures	Presurgery	Postsurgery
Serum immunoglobulin G		
Yoga mean ± (S.D)	5.6 ± 3.6	5.9 ± 3.8
Control mean ± (S.D)	5.1 ± 2.9	7.3 ± 5.2*
⇕Adjusted mean change scores (y-c)		
(95% CI)		-1.5 ± 1.1
		-3.6 to 0.69
Serum immunoglobulin M		
Yoga mean ± (S.D)	0.8 ± 0.5	0.9 ± 0.7
Control mean ± (S.D)	0.8 ± 0.5	1.0 ± 0.7
⇕Adjusted mean change scores (y-c)		
(95% CI)		-0.2 ± 0.2
		-0.5 to 0.2
Serum immunoglobulin A		
Yoga 33 mean ± (S.D)	1.1 ± 0.95	1.13 ± 0.7
Control 32 mean ± (S.D)	1.2 ± 1.05	1.84 ± 1.23**
⇕Adjusted mean change scores (y-c)		
(95% CI)		-0.765 ± 0.24**
		-1.24 to -0.29

* *P* < 0.05

** *P* < 0.01

***P < 0.001, y = Yoga, c = Control/ Supportive therapy group

*P* values for paired † test in yoga and control groups

⇕ Posttest scores (y-c) adjusted for their baseline scores between yoga and control groups with 95% CI and using ANCOVA for *P* values

Subjects' serum samples were assessed for Immunoglobulins G, M and A at baseline following surgery. A paired samples t test showed a significant increase in IgA levels following surgery in the control group [t (32) = -3.2, *P* = 0.005, 95% CI (-1.1 to -0.21] but no significant changes in the yoga group. Analysis of covariance using the baseline presurgery measure as a covariate also showed a significant decrease in IgA levels following surgery in the yoga group [F (62)=10.21, *P* =0.001] as compared to the controls. There was no significant within- and between-group changes in IgM and IgG levels.

#### Lymphocyte subsets [Table 5]

**Table 5:** Comparison of Lymphocyte subsets CD4, CD8 and CD56 % populations in yoga and control groups following surgery using paired t test and ANCOVA

Outcome measures Lymphocyte subsets in %	Presurgery	Postsurgery
CD4+ counts in %		
Yoga mean ± (S.D)	38.9 ± 6.1	35.3 ± 6.4*
Control mean ± (S.D)	38.3 ± 6	34.8 ± 5.1*
⇕Adjusted mean change scores (y-c)
(95% CI)		0.5 ± 1.4
		-2.3 to 3.3
CD8+ counts in %		
Yoga mean ± (S.D)	35 ± 5.9	33.1 ± 4.7
Control mean ± (S.D)	35.8 ± 6.6	32.1 ± 5.1**
⇕Adjusted mean change scores (y-c)
(95% CI)		1.3 ± 1.2
		-1.1 to 3.6
CD56+ counts in %		
Yoga mean (S.D)	19.7 ± 6.8	20.4 ± 4.9
Control mean (S.D)	21.8 ± 8.4	17.5 ± 4.3**
⇕ Adjusted mean change scores (y-c)		2.8 ± 1.2 *
(95% CI)		0.4 to 5.1

* *P* < 0.05

** *P* < 0.01

***P < 0.001, y = Yoga, c = Control/Supportive therapy group

*P* values for paired † test in yoga and control groups

⇕ Posttest scores (y-c) adjusted for their baseline scores between yoga and control groups with 95% CI and using ANCOVA for *P* values

T Lymphocyte subsets such as CD4+, CD8+ and CD56+ % were assessed before and after four weeks after surgery. A paired samples t test done to assess the changes in CD56 % following surgery within groups showed a significant decrease in CD56 % following surgery in the control group [t (32) = 3.57, *P* = 0.001), 95% CI (1.85 to 6.76)] but not in the yoga group. Analysis of variance done on these post- measures using the baseline measure of CD56 % as a covariate showed significantly higher levels of CD56 % in the yoga group following surgery [F (62) = 5.78, *P* = 0.019] as compared to the controls. A paired samples t test done to assess the changes in CD4 % following surgery within groups showed a significant decrease in CD4 % counts following surgery in the control group [t (35)= 3.31, *P* = 0.002, 95% CI (1.62 to 6.72)] but not in the yoga group. Analysis of variance on post- measures using baseline measures of CD4 % as a covariate did not show any significant changes between groups following surgery. A paired samples t test done to assess the changes in CD8 % following surgery within groups showed a significant decrease in CD8 % following surgery in the control group [t (34) = 3.46, *P* = 0.001, 95% CI (1.64 to 6.30)] but not in the yoga group. Analysis of variance on this post- measure using the baseline measure of CD8 % as a covariate did not show any significant changes between groups following surgery.

## DISCUSSION

The results suggest a significant decrease in psychological morbidity such as anxiety state and trait, depression, treatment-related symptoms and improvement in the quality of life in the yoga group as compared to the controls following surgery. There was also a significantly lower decrease in CD56 % in the yoga group as compared to the controls and lower levels of serum IgA in the yoga group as compared to controls postoperatively. However, there was a significant decrease in CD4, CD8 and CD56 % in the control group alone following surgery.

Although studies have shown various stress reduction interventions to modulate serum IgA levels and lymphocyte subsets in individuals, the fact that yoga helped decrease IgA levels postoperatively could be confounded by the heterogeneity in the extent of disease, type of surgery and disease stage among the groups. This is because earlier studies have shown that the tumor load is directly related to the serum IgA levels in breast cancer patients.[46] On the contrary, it can also be argued that yoga also helped reduce stress and improved anti-tumor immune responses that could have facilitated this change.

Although there is a dearth of literature using yoga interventions postsurgery, the beneficial effects conferred by similar stress reduction interventions nevertheless support our findings.[25,47] Psychotherapeutic intervention studies have used a number of diverse strategies which have positively affected immune function including hypnosis, relaxation, exercise, classical conditioning, self-disclosure, exposure to a phobic stressor to enhance perceived coping self-efficacy and cognitive-behavioral therapies.[20] A variety of hypnotic-relaxation intervention appear to shorten hospital stays, decrease pain and promote faster recovery following surgery[48] and most are brief, often single sessions and many involve taped suggestions. For example, 241 patients undergoing a stressful medical procedure were randomized to receive peri-operative standard care, structured attention or self-hypnotic relaxation. Self-hypnotic relaxation patients showed decreased pain and anxiety, lower use of self-administered pain medication, shorter procedure times and less hemodynamic instability than the other two groups.[49] These group differences were particularly impressive in view of the brevity of the intervention and the presumed heterogeneity of the patients' hypnotizable abilities. Another study showed that greater increases in relaxation in response to the intervention were associated with higher NK cell numbers and activity in healthy students taking medical exams.[50]

In contrast to the above relatively mild and predictable stress of examinations, breast cancer surgery is a high-stakes stressor with possible consequences that include death, pain, disfigurement, economic losses, alteration in social roles and uncertainty about the outcome.[24] Similar to other studies,[51] patients in our study displayed heightened anxiety prior to surgery and heightened distress persisting in the postoperative period. Studies have shown that if these stressors are perceived as uncontrollable and unpredictable, they can continue to be associated with elevated stress hormones.[52] Studies in both animal models and humans have shown elevations in plasma levels of epinephrine and cortisol to reflect sympathetic nervous system activation and hypothalamo-pituitary axis activation.[53,54] Coincidentally, such activation is also known to suppress NK cell activity,[15] reduce lymphocyte proliferative responses to mitogens and bring about changes in lymphocyte subpopulations such as NK cell counts.[55] Such immune suppression following surgery in cancer patients has also been implicated in the promotion of metastasis via numerous mechanisms including the suppression of natural killer cell activity and counts by stress hormones.[16,56] The ability to “unwind” after stressful encounters, i.e., a quicker return to one's neuroendocrine baseline, influences the total burden that the stressors place on the individual.[57] Earlier studies have advocated that interventions promoting early adaptation can produce substantial benefits for mental and physical health.[53] Our intervention was helpful in reducing postoperative distress and anxiety and also helped improve immune responses in terms of changes in lymphocyte subpopulations such as CD4 and CD8% and NK cell counts postoperatively indicating that it helped promote adaptation to this stressor. The changes in lymphocyte subpopulations seen with yoga interventions could be attributed to stress reduction effects and adaptation to the stress of surgery that could have facilitated a decrease in postoperative distress and consequent improvement in immune outcomes. Catecholamines and glucocorticoids have been shown to rapidly and markedly affect the distribution of NK cells among different immune compartments (e.g., spleen, liver, lungs, circulating blood, marginating pool of blood, etc)[58,59] and it may be hypothesized that changes in these hormone levels could be one of the mechanisms of action of our intervention.

Our study however, has several limitations: i) we assessed only NK cell number and T lymphocyte subsets and not NK cell function or T lymphocyte function, thereby studying the effect of stress on immune cell trafficking rather than immune cell function. ii) We assessed CD4, CD8 and NK cell % by immunohistochemistry as opposed to fluorescence-activated cell sorting (FACS) and this is a major limitation of our study. The tests were run in duplicate by a single observer and quality control was maintained by cross-verifying the NK cell counts with FACS for standardization purposes. Our values of NK cells in % are similar to earlier findings.[60,61] iii) As patients in this study were those who were enrolled to participate in a trial using yoga and supportive therapy intervention, it will be worthwhile to speculate that cancer patients who seek psychosocial interventions and care are different from those who don't in terms of psychological distress and immune outcomes.[62] Consequently, meta-analyses has shown that these groups would also benefit more from such interventions,[63] thereby limiting the generalizability of our findings.

The stress reduction and immune-enhancing benefits conferred by our intervention could have implications for breast cancer patients who have to endure long-term treatments that could cause more distress and immune suppression.[64] Although studies support the fact that even brief stress reduction interventions can have possible clinical benefits,[22] it remains to be seen if these benefits can be sustained significantly over a period. Although our study did not show any functional changes in immune responses (lymphocyte proliferation in response to antigens or NK cell activity), it nevertheless supports a trend for the prevention of immune suppression in terms of cell trafficking and counts. However, larger controlled trials with more advanced measures of immune function are needed to study the immediate effects of yoga intervention on surgery outcomes.
